# A Neutrophil‐Based Predictive Model for Axillary De‐Escalation After Neoadjuvant Therapy in Node‐Positive Breast Cancer

**DOI:** 10.1049/syb2.70046

**Published:** 2025-11-27

**Authors:** Exian Mou, Rui Guo, Huaichao Luo, Jia Xu, Wen Wei

**Affiliations:** ^1^ Department of Plastic and Reconstructive Surgery Sichuan Clinical Research Center for Cancer Sichuan Cancer Hospital & Institute Sichuan Cancer Center Affiliated Cancer Hospital of University of Electronic Science and Technology of China Chengdu China; ^2^ Department of Clinical Laboratory Sichuan Clinical Research Center for Cancer Sichuan Cancer Hospital & Institute Sichuan Cancer Center University of Electronic Science and Technology of China Chengdu China; ^3^ Department of Medical Oncology Sichuan Clinical Research Center for Cancer Sichuan Cancer Hospital & Institute Sichuan Cancer Center University of Electronic Science and Technology of China Chengdu China

**Keywords:** blood, cancer, Pharmacology, toxicology

## Abstract

This study aimed to develop a novel immunoscore system integrating peripheral blood immune signatures and clinical factors to predict axillary pathological complete response (apCR) in clinically node‐positive (cN+) breast cancer patients after neoadjuvant treatment (NAT) and facilitate personalized axillary de‐escalation strategies. A retrospective analysis was conducted on cN+ breast cancer patients who received NAT at Sichuan Cancer Hospital, with 437 cases (June 2018–June 2023) as the training set and 266 cases (July 2023–July 2024) as the validation set, where clinicopathological data and peripheral blood immune indices were collected, multivariate logistic regression was used to identify independent predictors of apCR, predictive models were compared via ROC analysis, and a nomogram was constructed based on the optimal model. The apCR rate was 48.7% (213/437), with multivariate analysis revealing HER2 positivity (OR = 6.32, 95% CI: 3.95–10.12, *p* < 0.001), clinical response (RECIST 1.1), and baseline neutrophil count (OR = 1.26 per unit increase, 95% CI: 1.08–1.48, *p* = 0.003) as independent predictors, while the combined clinical‐hematologic model (AUC = 0.766) outperformed the clinical‐only model (AUC = 0.757) with consistent performance in the validation cohort (AUC = 0.759) and baseline neutrophil count exhibiting a strong linear correlation with apCR rates (*r* = 0.97, *p* < 0.001). In conclusion, baseline neutrophil count, HER2 status, and clinical response jointly predict apCR post‐NAT in cN+ breast cancer, and the proposed immunoscore nomogram offers a practical tool to guide axillary de‐escalation and optimize surgical decision‐making.

## Graphical abstract

Purpose: This study developed a novel immunoscore combining peripheral blood immune markers and clinical factors to predict axillary pathological complete response (apCR) in clinically node‐positive breast cancer after neoadjuvant therapy, enabling personalised axillary de‐escalation. Key findings: HER2 positivity, clinical tumour response (RECIST 1.1) and elevated baseline neutrophil count (showing strong linear correlation with apCR, ∗*r*∗ = 0.97 and *p* < 0.001) were independent predictors of apCR, with the combined clinical‐haematologic model achieving AUC = 0.766. Conclusion: The validated immunoscore nomogram integrates these predictors to optimise surgical decision‐making for axillary management.

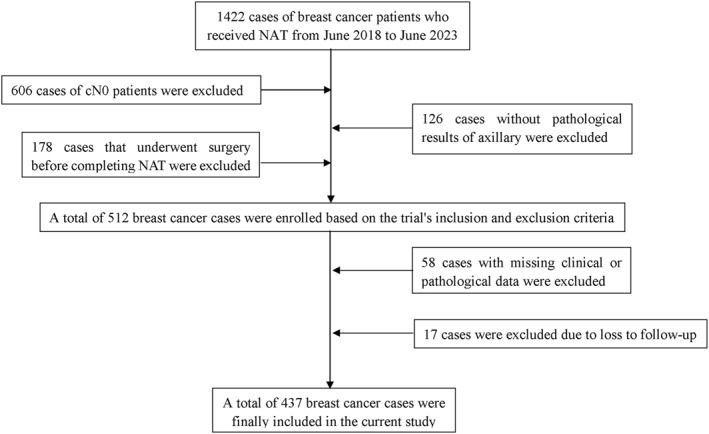

## 1 Introduction

Breast cancer is the second most common cancer globally and the fourth leading cause of cancer‐related deaths [1]. It is the most prevalent malignancy among women worldwide [2]. Sentinel lymph node biopsy (SLNB) is a critical tool for accurately assessing axillary lymph node status in patients with early‐stage breast cancer. By avoiding unnecessary axillary lymph node dissection (ALND), SLNB significantly reduces postoperative complications such as upper limb oedema. Consequently, SLNB has become the standard approach for axillary lymph node staging in clinically node‐negative (cN0) breast cancer cases [3].

As the indications for neoadjuvant treatment (NAT) in breast cancer continue to expand, the use of SLNB after NAT in patients with early‐stage breast cancer who transition from clinically node‐positive (cN+) to cN0 status has become a key research focus [4]. However, current techniques face several limitations, including the difficulty in obtaining radioactive tracers, the technical complexity of positive lymph node labelling, high costs and challenges in controlling the number of detected sentinel lymph node (SLN). These issues hinder the widespread clinical adoption of these methods [5, 6].

Predicting axillary pathological complete response (apCR) following NAT holds substantial clinical significance. Patients who achieve apCR generally have a better prognosis compared to those who do not, and they are ideal candidates for SLNB, allowing them to safely avoid ALND [7]. Although current research has explored various approaches, including clinical factors, radiomics, high‐throughput sequencing data analysis and multigene signatures with some promising results, there is currently no reliable method for accurately predicting apCR [7, 8, 9, 10, 11, 12].

Peripheral blood immune indices have gained considerable attention in breast cancer research as key markers of systemic immune‐inflammatory status, including leucocyte, monocyte, neutrophil and lymphocyte counts, as well as derived ratios such as the neutrophil‐to‐lymphocyte ratio (NLR), platelet‐to‐lymphocyte ratio (PLR) and lymphocyte‐to‐monocyte ratio (LMR) [13, 14]. In triple‐negative breast cancer (TNBC), higher lymphocyte counts correlate with improved long‐term survival, whereas lower NLR predicts increased pathological complete response (pCR) rates after NAT [15]. These indices also show prognostic and predictive value in HER2‐positive disease, though their utility remains limited in Luminal‐type breast cancer [16]. However, prior studies have been constrained by small sample sizes, inconsistent findings and a predominant focus on total pCR (tpCR) or breast pCR (bpCR), with notably scarce data on their ability to predict apCR in patients with cN+ receiving NAT.

To address these knowledge gaps, we aim to (1) identify peripheral blood immune signatures that predict apCR; (2) develop a combined clinical‐haematologic predictive model and (3) establish an immunoscoring system to guide SLNB decision‐making in patients with cN+ after NAT. Ultimately, this work seeks to enable precision surgical strategies while ensuring oncological safety.

## 2 Materials and Methods

### 2.1 Study Design and Participants

This retrospective study (Ethics approval: KY‐2022‐051 and Clinical trial registration: ChiCTR2300070356) analysed patients with cN+ breast cancer who received NAT at Sichuan Cancer Hospital, with those treated between June 2018 and June 2023 as the training set and cases from July 2023 to July 2024 as the validation set. Eligible patients met all inclusion criteria: (1) Women aged 18–70 years; (2) biopsy‐confirmed invasive breast cancer with axillary node metastasis; (3) no distant metastasis or prior systemic therapy: (4) completed NAT (anthracyclines/taxanes ± anti‐HER2 therapy); (5) underwent post‐NAT ALND and (6) complete clinical data available. Exclusion criteria: pregnancy, inflammatory breast cancer or prior axillary surgery.

### 2.2 Collection and Assessment Criteria of Clinical Characteristics

Clinical data of enrolled patients were retrieved from the Breast Cancer Database of Sichuan Cancer Hospital, including four categories of information:

1. Demographic information: Name, gender, age, hospital number, height, weight and body mass index (BMI).
2. Clinicopathological information: Histological grade of breast cancer, clinical T (cT) stage, clinical N (cN) stage, expression of oestrogen receptor (ER), progesterone receptor (PR), human epidermal growth factor receptor 2 (HER2) and Ki‐67.
3. Treatment information: NAT regimens and anti‐HER2 targeted therapy regimens for HER2‐positive breast cancer.

NAT regimens were formulated in accordance with recommendations from authoritative guidelines, including the NCCN Guidelines and the Guidelines for the Diagnosis and Treatment of Breast Cancer by the Chinese Anti‐Cancer Association. All patients received NAT regimens based on anthracyclines and/or taxanes. Patients with HER2‐positive completed targeted therapy with trastuzumab alone or trastuzumab combined with pertuzumab.

4. Efficacy evaluation after NAT: Efficacy was assessed by colour Doppler ultrasound and relevant data were recorded. Clinical response was evaluated according to the Response Evaluation Criteria in Solid Tumours (RECIST 1.1), categorised into four grades: complete response (CR), partial response (PR), progressive disease (PD) and stable disease (SD).
5. Pathological assessment criteria for axillary nodes after NAT: All pathological evaluations were conducted and reviewed by at least two pathologists with the title of attending physician or above. ApCR was defined as the absence of residual tumour cells in all axillary lymph nodes; micrometastases and isolated tumour cells (ITCs) were both considered nonachievement of apCR.

### 2.3 Collection of Peripheral Blood Immune Indices

Laboratory data of enrolled patients were obtained from the hospital information system. The haematology analyser used for blood tests was the Mindray Automated Haematology Analyser Model BC‐10. The timing window for blood tests before NAT was set at 1 month prior to treatment, whereas the window for tests after NAT and before surgery was 1 month following NAT completion. Baseline neutrophil counts were measured in accordance with the local laboratory standards of the clinical laboratory. The normal reference range was 2.0–7.5 × 10^9^/L, which is consistent with international clinical guidelines. Peripheral blood immune indices were collected from routine blood tests and biochemical results at two time points: before NAT (upon initial admission, prior to any antitumour treatment) and before surgery after completion of NAT. The indices included WBC, neutrophil, lymphocyte, monocyte and platelet counts; NLR, PLR and LMR.

### 2.4 Statistical Analysis

All statistical analyses were performed using SPSS version 24.0 (SPSS Inc., Chicago, IL, USA). Continuous variables were expressed as mean ± standard deviation (*x̅* ± *s*), and differences between groups were compared using independent samples *t*‐test. Categorical variables were presented as counts (percentages), and comparisons were made using the chi‐squared test. Factors with statistical significance in univariate analysis were included in a logistic regression model for stepwise regression analysis to calculate odds ratios (OR) and 95% confidence intervals (CIs). The receiver operating characteristic (ROC) curve and area under the curve (AUC) were used to evaluate the overall performance of the model. A two‐tailed *p*‐value < 0.05 was considered statistically significant. The AUC ranges from 0.5 to 1, with higher values indicating better predictive performance of the model.

## 3 Results

### 3.1 Baseline Characteristics

**FIGURE 1:**
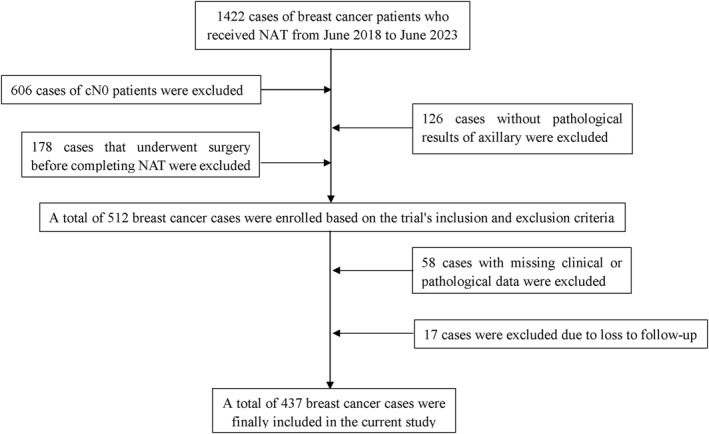
Flowchart of patient enrollment in the study. cN0, clinically node‐negative; NAT, neoadjuvant treatment.

According to the inclusion and exclusion criteria, a total of 437 breast cancer patients were enrolled in this study, with the specific process detailed in Figure 1, and the baseline characteristics of these patients were showed in Table 1. The cohort had a mean age of 50.09 ± 9.99 years and a mean BMI of 24.32 ± 3.22 kg/m^2^. Clinical staging showed a predominance of T2 (320 cases, 73.2%) and N1 (289 cases, 66.1%) disease. Regarding molecular subtypes, 297 cases (68.0%) were ER‐positive, 264 cases (60.4%) were PR‐positive and 151 cases (34.6%) were HER2‐positive. A high Ki‐67 index (≥ 20%) was observed in 352 cases (80.5%). Pathological evaluation revealed that 95 cases (21.7%) achieved tpCR, 124 cases (28.4%) achieved bpCR and 213 cases (48.7%) achieved apCR.

**TABLE 1:** Patient and tumour characteristics.

Characteristic	All *n* = 437	apCR *n* = 213	Non‐apCR *n* = 224
Age (years, $\bar{x}$ ± *s*)	50.09 ± 9.99	49.29 ± 9.17	50.85 ± 10.68
BMI^a^ ($\bar{x}$ ± *s*)	24.32 ± 3.22	24.29 ± 3.21	24.35 ± 3.24
cT^b^			
0	2 (0.5%)	1 (0.5%)	1 (0.4%)
1	31 (7.1%)	12 (5.6%)	19 (8.5%)
2	320 (73.2%)	166 (77.9%)	154 (68.8%)
3	59 (13.5%)	25 (11.7%)	34 (15.2%)
4	25 (5.7%)	9 (4.2%)	16 (7.1%)
cN^b^			
1	289 (66.1%)	145 (68.1%)	144 (64.3%)
2	74 (16.9%)	35 (16.4%)	39 (17.4%)
3	74 (16.9%)	33 (15.5%)	41 (18.3%)
Histological grade			
I–II	216 (49.4%)	91 (42.7%)	125 (55.8%)
III	107 (24.5%)	65 (30.5%)	42 (18.8%)
NA	114 (26.1%)	57 (26.8%)	57 (25.4%)
ER			
Negative	140 (32.0%)	86 (40.4%)	54 (24.1%)
Positive	297 (68.0%)	127 (59.6%)	170 (75.9%)
PR			
Negative	173 (39.6%)	105 (49.3%)	68 (30.4%)
Positive	264 (60.4%)	108 (50.7%)	156 (69.6%)
HER2			
Negative	286 (65.4%)	97 (45.5%)	189 (84.4%)
Positive	151 (34.6%)	116 (54.5%)	35 (15.6%)
Ki67^a^			
< 20%	84 (19.2%)	32 (15.0%)	52 (23.2%)
≥ 20%	352 (80.5%)	180 (84.5%)	172 (76.8%)
NAC regimen			
A + T	353 (80.8%)	148 (69.5%)	205 (91.5%)
T	84 (19.2%)	65 (30.5%)	19 (8.5%)
Clinical response^c^			
CR	13 (3.0%)	11 (5.2%)	2 (0.9%)
PR	307 (70.3%)	165 (77.5%)	142 (63.4%)
SD	107 (24.5%)	32 (15.0%)	75 (33.5%)
PD	10 (2.3%)	5 (2.3%)	5 (2.2%)

*Note:* A + T, anthracycline‐taxane combination regimen.

Abbreviations: A, anthracycline; ApCR, axillary pathological complete response; cN, clinical N category; cT, clinical T category; ER, Estrogen receptor; HER2, human epidermal growth factor receptor 2; NAC, neoadjuvant chemotherapy; PR, progesterone receptor; T, taxanes.

^a^ Data on BMI and Ki‐67 were missing for one case each.

^b^ T and N category were assessed using the 8th edition of American Joint Committee on Cancer TNM staging system.

^c^ Clinical response was evaluated according to the Response Evaluation Criteria in Solid Tumours (RECIST) version 1.1.

### 3.2 Univariate and Multivariate Analysis of Clinical Characteristics Associated With apCR

Univariate analysis identified significant associations between apCR and histological grade (*p* = 0.007), ER status (*p* < 0.001), PR status (*p* < 0.001), HER2 status (*p* < 0.001), Ki‐67 index (*p* = 0.032), NAC regimen (*p* < 0.001) and clinical response (*p* < 0.001) (Table 2). Patients with grade III tumours, ER‐negative status, PR‐negative status, HER2‐positive status, high Ki‐67 (≥ 20%), taxane‐based regimens (T) and complete clinical response (CR) showed higher ApCR rates.

**TABLE 2:** Univariate and multivariate logistic regression for clinical characteristics associated with ApCR.

Characteristic	apCR *n* = 213	Non‐apCR *n* = 224	Univariate		Multivariate	
			Statistical value	*p*	OR (95% CI)	*p*
Age (years, $\bar{x}$ ± *s*)	49.29 ± 9.17	50.85 ± 10.68	*t* = 1.643	0.101		
BMI^a^ ($\bar{x}$ ± *s*)	24.29 ± 3.21	24.35 ± 3.24	*t* = 0.186	0.852		
cT			*χ* ^2^ = 5.267	0.237		
0	1 (50.0%)	1 (50.0%)				
1	12 (38.7%)	19 (61.3%)				
2	166 (51.9%)	154 (48.1%)				
3	25 (42.4%)	34 (57.6%)				
4	9 (36.0%)	16 (64.0%)				
cN			*χ* ^2^ = 0.808	0.668		
1	145 (50.2%)	144 (49.8%)				
2	35 (47.3%)	39 (52.7%)				
3	33 (44.6%)	41 (55.4%)				
Histological grade			*χ* ^2^ = 10.025	0.007		0.198
I–II	91 (42.1%)	125 (57.9%)				
III	65 (60.7%)	42 (39.3%)				
NA	57 (50.0%)	57 (50.0%)				
ER			*χ* ^2^ = 13.271	< 0.001		0.588
Negative	86 (61.4%)	54 (38.6%)				
Positive	127 (42.8%)	170 (57.2%)				
PR			*χ* ^2^ = 16.374	< 0.001		0.038
Negative	105 (60.7%)	68 (39.3%)			1.00	
Positive	108 (40.9%)	156 (59.1%)			0.63 (0.40–0.98)	
HER2			*χ* ^2^ = 72.814	< 0.001		< 0.001
Negative	97 (33.9%)	189 (66.1%)			1.00	
Positive	116 (76.8%)	35 (23.2%)			5.52 (3.44–8.86)	
Ki67^a^			*χ* ^2^ = 4.617	0.032		0.226
< 20%	32 (38.1%)	52 (61.9%)				
≥ 20%	180 (51.1%)	172 (48.9%)				
NAC regimen			*χ* ^2^ = 34.139	< 0.001		0.072
A + T	148 (41.9%)	205 (58.1%)				
T	65 (77.4%)	19 (22.6%)				
Clinical response			*χ* ^2^ = 25.395	< 0.001		< 0.001
CR	11 (84.6%)	2 (15.4%)			1.00	
PR	165 (53.7%)	142 (46.3%)			0.24 (0.05–1.16)	0.076
SD	32 (29.9%)	75 (70.1%)			0.09 (0.02–0.45)	0.004
PD	5 (50.0%)	5 (50.0%)			0.16 (0.02–1.29)	0.085

*Note:* A + T, anthracycline‐taxane combination regimen.

Abbreviations: A, anthracycline; ApCR, axillary pathological complete response; BMI, body mass index; CI, confidence interval; cN, clinical N category; cT, clinical T category; ER, Estrogen receptor; HER2, human epidermal growth factor receptor 2; NAC, neoadjuvant chemotherapy; OR, odds ratio; PR, progesterone receptor; T, taxanes.

^a^ Data on BMI and Ki‐67 were missing for one case each.

Multivariate analysis confirmed HER2 positivity (OR = 5.52, 95% CI: 3.44–8.86 and *p* < 0.001) and PR negativity (OR = 0.63, 95% CI: 0.40–0.98 and *p* = 0.038) as independent predictors of ApCR. Notably, patients achieving clinical CR demonstrated substantially higher ApCR likelihood (reference) compared to those with PR (OR = 0.24 and *p* = 0.076), SD (OR = 0.09 and *p* = 0.004) or PD (OR = 0.16 and *p* = 0.085). The predictive value of histological grade and Ki‐67 index observed in univariate analysis was not retained in multivariate models (*p* = 0.198 and *p* = 0.226, respectively).

### 3.3 Univariate and Multivariate Analysis of Clinical Factors and Peripheral Blood Immune Indices Associated With apCR

Univariate analysis demonstrated significant associations between ApCR and multiple baseline peripheral blood indices: higher WBC count (6.13 ± 1.62 vs. 5.81 ± 1.62 and *p* = 0.045) and neutrophil count (4.08 ± 1.41 vs. 3.76 ± 1.36 and *p* = 0.014) were observed in the ApCR group, with NLR showing borderline significance (2.82 ± 1.30 vs. 2.56 ± 1.41 and *p* = 0.051) (Table 3).

**TABLE 3:** Univariate logistic regression for peripheral blood immune indices associated with ApCR.

Peripheral blood immune indices	*n*	$\bar{x}$ ± *s*	apCR		Non‐apCR		Statistical value	*p*
			*n*	$\bar{x}$ ± *s*	*n*	$\bar{x}$ ± *s*		
Baseline (pre‐NAT)^a^								
WBC	434	5.97 ± 1.63	213	6.13 ± 1.62	221	5.81 ± 1.62	*t* = −2.011	0.045
Neutrophil	434	3.92 ± 1.39	213	4.08 ± 1.41	221	3.76 ± 1.36	*t* = −2.455	0.014
Lymphocyte	434	1.61 ± 0.54	213	1.59 ± 0.51	221	1.63 ± 0.57	*t* = 0.822	0.412
Monocyte	434	0.31 ± 0.12	213	0.32 ± 0.10	221	0.30 ± 0.14	*t* = −0.892	0.373
Platelet	434	212.65 ± 63.21	213	212.91 ± 58.31	221	213.09 ± 67.74	*t* = 0.145	0.885
NLR	434	2.69 ± 1.36	213	2.82 ± 1.30	221	2.56 ± 1.41	*t* = −1.955	0.051
PLR	434	144.57 ± 69.01	213	144.37 ± 55.75	221	144.76 ± 79.64	*t* = 0.057	0.955
LMR	434	5.73 ± 3.81	213	5.66 ± 5.00	221	5.81 ± 2.14	*t* = 0.419	0.675
Preoperative (post‐NAT)^a^								
WBC	408	5.58 ± 2.95	198	5.42 ± 2.43	210	5.73 ± 3.38	*t* = 1.067	0.287
Neutrophil	408	3.93 ± 2.78	198	3.78 ± 2.21	210	4.07 ± 3.22	*t* = 1.032	0.303
Lymphocyte	408	1.19 ± 0.42	198	1.18 ± 0.42	210	1.19 ± 0.42	*t* = 0.411	0.682
Monocyte	408	0.37 ± 0.17	198	0.36 ± 0.18	210	0.37 ± 0.16	*t* = 0.108	0.914
Platelet	408	212.73 ± 67.38	198	209.78 ± 66.91	210	215.51 ± 67.86	*t* = 0.858	0.391
NLR	408	3.64 ± 2.72	198	3.50 ± 2.10	210	3.77 ± 3.20	*t* = 1.017	0.310
PLR	408	198.16 ± 89.75	198	197.61 ± 89.38	210	198.67 ± 90.30	*t* = 0.119	0.906
LMR	408	4.24 ± 10.72	198	4.82 ± 15.29	210	3.69 ± 1.68	*t* = −1.07	0.285

Abbreviations: ApCR, axillary pathological complete response; LMR, lymphocyte‐to‐monocyte ratio; NLR, neutrophil‐to‐lymphocyte ratio; PLR, platelet‐to‐lymphocyte ratio; WBC, white blood cell.

^a^ Data on baseline peripheral blood immune indices were missing for three case, and data on preoperative peripheral blood immune indices were missing for three case.

Multivariate analysis confirmed HER2 positivity (OR = 6.32, 95% CI: 3.95–10.12 and *p* < 0.001) and elevated baseline neutrophil count (OR = 1.26 per unit increase, 95% CI: 1.08–1.48 and *p* = 0.003) as independent predictors. PR‐negative status showed marginal significance (OR = 0.63, 95% CI: 0.40–0.98 and *p* = 0.053), while complete clinical response remained strongly predictive (reference) versus SD (OR = 0.08, 95% CI: 0.02–0.40 and *p* = 0.002). Notably, the predictive value of WBC count and histological grade identified in univariate analysis was attenuated in the adjusted model (*p* > 0.05) (Table 4).

**TABLE 4:** Multivariate logistic regression for clinical factors and peripheral blood immune indices associated with ApCR.

Characteristic	apCR *n* = 213	Non‐apCR *n* = 224	Univariate		Multivariate	
			Statistical value	*p*	OR (95% CI)	*p*
ER			*χ* ^2^ = 13.271	< 0.001		0.106
Negative	86 (61.4%)	54 (38.6%)				
Positive	127 (42.8%)	170 (57.2%)				
PR			*χ* ^2^ = 16.374	< 0.001		0.053
Negative	105 (60.7%)	68 (39.3%)			1.00	
Positive	108 (40.9%)	156 (59.1%)			0.63 (0.40–0.98)	
Histological grade			*χ* ^2^ = 10.025	0.007		0.078
I–II	91 (42.1%)	125 (57.9%)				
III	65 (60.7%)	42 (39.3%)				0.026
NA	57 (50.0%)	57 (50.0%)				0.672
HER2			*χ* ^2^ = 72.814	< 0.001		< 0.001
Negative	97 (33.9%)	189 (66.1%)			1.00	
Positive	116 (76.8%)	35 (23.2%)			6.32 (3.95–10.12)	
Ki67^a^			*χ* ^2^ = 4.617	0.032		0.155
< 20%	32 (38.1%)	52 (61.9%)				
≥ 20%	180 (51.1%)	172 (48.9%)				
NAC regimen			*χ* ^2^ = 34.139	< 0.001		0.050
A + T	148 (41.9%)	205 (58.1%)				
T	65 (77.4%)	19 (22.6%)				
Clinical response			*χ* ^2^ = 25.395	< 0.001		< 0.001
CR	11 (84.6%)	2 (15.4%)			1.00	
PR	165 (53.7%)	142 (46.3%)			0.21 (0.04–1.04)	0.056
SD	32 (29.9%)	75 (70.1%)			0.08 (0.02–0.40)	0.002
PD	5 (50.0%)	5 (50.0%)			0.13 (0.02–1.02)	0.052
Neutrophil (baseline)^a^	213 (49.1%)	221 (50.9%)	*t* = −2.455	0.014	1.26 (1.08–1.48)	0.003

*Note:* A + T, anthracycline‐taxane combination regimen.

Abbreviations: A, anthracycline; ApCR, axillary pathological complete response; CI, confidence interval; ER, Estrogen receptor; HER2, human epidermal growth factor receptor 2; NAC, neoadjuvant chemotherapy; OR, odds ratio; PR, progesterone receptor; T, taxanes.

^a^ Ki‐67 data were unavailable for 1 patient; baseline neutrophil counts were missing for 3 patients.

### 3.4 Independent Predictive Value of Baseline Neutrophil Count for apCR

**FIGURE 2:**
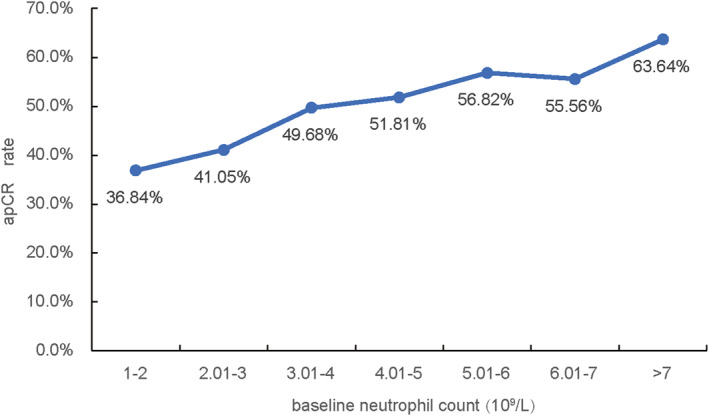
Correlation between baseline neutrophil count and apCR rate.

In our extended analysis, the “per unit increase” in the odds ratio (OR) was defined as a 1.0 × 10^9^/L elevation in neutrophil count (e.g., 2.0–3.0 × 10^9^/L). We further found a strong linear correlation between baseline neutrophil levels and apCR rates (*r* = 0.97 and *p* < 0.001), with neutrophil counts conforming to a normal distribution. Moreover, as the neutrophil count threshold increased, progressively higher apCR achievement rates were observed (Figure 2).

### 3.5 Development of Clinical‐apCR Prediction Model and Haematologic‐Clinical‐apCR Prediction Model

**FIGURE 3:**
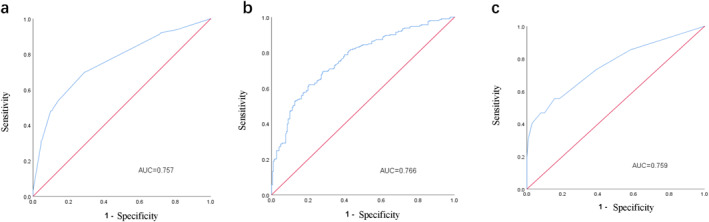
Clinical‐apCR prediction model (a); haematologic‐clinical‐apCR prediction model (training set) (b) and haematologic‐clinical‐apCR prediction model (validation set) (c).

The clinical prediction model was constructed using significant predictors (*p* < 0.05) from multivariate logistic regression analysis, including PR status, HER2 status and clinical treatment response. ROC curve analysis demonstrated that this model achieved an AUC of 0.757 (95% CI: 0.711–0.802), with a sensitivity of 70.0% and specificity of 71.0% (Figure 3a). The full logistic regression equation for Clinical‐apCR prediction model:logit (*p*) = 1.31–0.47*PR+1.71*HER2‐1.44*PR‐2.43*SD‐1.83*PD.

The combined clinical and peripheral blood immune indices model incorporated significant factors (*p* < 0.05) from multivariate analysis, including HER2 status, clinical treatment response and baseline neutrophil count. This integrated model showed improved predictive performance, with an AUC of 0.766 (95% CI: 0.722–0.811), sensitivity of 62.0% and specificity of 79.6% (Figure 3b). The full logistic regression equation for Haematologic‐Clinical‐apCR prediction model:logit (*p*) = 0.21 + 1.84*HER2‐1.56*PR‐2.58*SD‐2.08*PD+0.23* baseline neutrophil count.

**FIGURE 4:**
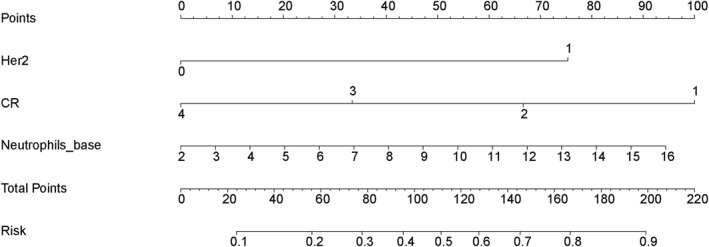
Nomogram based on the haematologic‐clinical‐apCR prediction model.

Using the superior‐performing integrated haematologic‐clinical‐apCR prediction model (higher AUC = 0.766 vs. 0.757), we developed a clinically applicable nomogram to generate an immunoscore for predicting apCR following NAT in patients with cN+ breast cancer (Figure 4).

### 3.6 Validation of the Haematologic‐Clinical‐apCR Prediction Model

The baseline characteristics of the training set (*n* = 434) and validation set (*n* = 266) were consistent (Table 5). In the validation set, the apCR rate was 46.6% (124/266) and the AUC of this prediction model was 0.759 (95% CI 0.700–0.817), with a sensitivity of 55.6% and a specificity of 84.4%, which further verified the accuracy of the model (Figure 3c).

**TABLE 5:** Comparison of baseline characteristics between the training set and the validation set.

Characteristic	Training set *n* = 434	Validation set *n* = 266	*p*
Age (years, $\bar{x}$ ± *s*)	50.08 ± 9.93	51.56 ± 9.58	0.128
BMI ($\bar{x}$ ± *s*)	24.31 ± 3.21	24.45 ± 3.21	0.946
cT			0.998
0	2 (0.5%)	1 (0.4%)	
1	30 (6.9%)	19 (7.1%)	
2	319 (73.5%)	192 (74.1%)	
3	59 (13.6%)	34 (12.8%)	
4	24 (5.5%)	15 (5.6%)	
cN			0.889
1	288 (66.4%)	183 (68.8%)	
2	74 (17.1%)	36 (13.5%)	
3	72 (16.6%)	47 (17.7%)	
Histological grade			0.674
I–II	216 (49.8%)	143 (53.8%)	
III	106 (24.4%)	55 (20.7%)	
NA	112 (25.8%)	68 (25.6%)	
ER			0.653
Negative	140 (32.3%)	78 (29.3%)	
Positive	294 (67.7%)	188 (70.7%)	
PR			0.948
Negative	173 (39.9%)	101 (38.0%)	
Positive	261 (60.1%)	165 (62.0%)	
HER2			0.970
Negative	283 (65.2%)	178 (66.9%)	
Positive	151 (34.8%)	88 (33.1%)	
Ki67			0.991
< 20%	83 (19.2%)	53 (19.9%)	
≥ 20%	350 (80.8%)	213 (80.1%)	

Abbreviations: BMI, body mass index; cN, clinical N category; cT, clinical T category; ER, Estrogen receptor; HER2, human epidermal growth factor receptor 2; PR, progesterone receptor.

## 4 Discussion

In this retrospective study involving 437 patients with  cN+ breast cancer who received standard NAT, we systematically assessed the associations between clinicopathological characteristics/peripheral blood immune parameters and apCR. Multivariable analysis identified four independent predictors of apCR: HER2 status (positive vs. negative), clinical treatment response (evaluated by RECIST criteria), PR status (negative vs. positive) and baseline neutrophil count. Notably, among all peripheral blood immune indices, baseline neutrophil count stood out as the most significant haematologic biomarker for predicting the likelihood of apCR.

In the present study, the apCR rate in patients with HER2‐positive breast cancer reached 76.8% (116/151), which was significantly higher than that in HER2‐negative patients (33.9%, 97/286) (*p* < 0.001). HER2 positivity was identified as an independent predictor of apCR, and this result is consistent with previous research findings [8, 17]. A study analysing factors related to apCR in HER2‐positive breast cancer included 215 patients with  cN+ HER2‐positive breast cancer, all of whom received dual‐targeted therapy. The overall apCR rate was 76.7%, with patients with HER2 3+ achieving a significantly higher rate (80.7%) than HER2 2+/FISH+ counterparts (63.3%, *p* = 0.011) [18]. Therefore, HER2 positivity can serve as an important reference factor for predicting apCR and implementing axillary de‐escalation surgery. Meanwhile, further attention can be paid to patients with HER2 3+ as they have a higher probability of achieving apCR.

Our data suggest an apparent linear decline in apCR rates across RECIST categories; among these, patients achieving CR or PR had superior apCR rates compared to those with SD (*p* < 0.001). Notably, the 50.0% apCR rate observed in patients with PD did not follow this continuum, likely reflecting the limited sample size of PD cases in our cohort (*n* = 10). Utilising clinical efficacy assessment to predict apCR represents a practical approach in clinical practice. The SN FNAC substudy provides compelling evidence for imaging‐based axillary assessment post‐NAT. Among patients classified as cN+ by ultrasound after NAT, 82.5% (47/57) were confirmed as ypN+ at surgery. Importantly, ultrasound‐reclassified patients with cN0 undergoing SLNB demonstrated significantly improved diagnostic accuracy, with false‐negative rates decreasing from 8.4% to 2.7% [19].

Our study demonstrates that baseline neutrophil count emerges as the most significant haematologic predictor of axillary pathological complete response (apCR) among peripheral blood immune indices. However, no significant associations with apCR were observed for other peripheral blood immune indices reported in previous literature to be potentially related to apCR, such as lymphocyte count, monocyte count, platelet count, NLR, PLR and LMR. Neutrophils in the breast cancer immune microenvironment are associated with poor prognosis. Inflammatory factors produced by tumours (e.g., IL‐8 and CXC chemokines) can recruit neutrophils into the tumour microenvironment [20]. Once in the tumour microenvironment, neutrophils polarise into a protumour phenotype and promote tumour cell proliferation by secreting neutrophil elastase, which activates the PI3K and PDGFR signalling pathways [21]. Meanwhile, basic research has shown that neutrophils can promote breast cancer progression by adhering to TNBC cells via CD11b‐ICAM1 [22]. Neutrophils play an important role in the immune microenvironment of TNBC and can serve as a significant prognostic marker for TNBC [23]. Currently, clinical trials are evaluating the efficacy of neutrophil‐targeted therapies in breast cancer, including Xiufuxin (NCT02370238) and tigatuzumab (NCT01307891). Neutrophils are expected to become a new therapeutic target in breast cancer, which supports our research findings [24].

In conclusion, HER2 status, clinical treatment response and baseline neutrophil count are robust predictors of apCR. Among peripheral blood immune indices, baseline neutrophil count stands out as the most potent biological marker for apCR prediction. The immunoprediction model and scoring system developed based on these parameters exhibit clinical utility in accurately identifying patients with a high probability of achieving apCR, thereby facilitating personalised axillary surgical de‐escalation following NAT.

## Author Contributions

**Exian Mou:** conceptualization, investigation, writing – original draft. **Rui Guo:** methodology, formal analysis, data curation. **Huaichao Luo:** software, validation, visualization. **Jia Xu:** supervision, project administration, writing – review and editing. **Wen Wei:** resources, supervision, writing – review and editing.

## Funding

The authors have nothing to report.

## Conflicts of Interest

The authors declare no conflicts of interest.

## Data Availability

The data that support the findings of this study are available from the corresponding author upon reasonable request.
